# A Capacity Audit of Medical Geneticists and Genetic Counsellors in South Africa, 2024: A National Crisis

**DOI:** 10.3390/genes15091173

**Published:** 2024-09-06

**Authors:** Marianne C. M. Gomes, Byron J. Gomes, Arnold L. Christianson, Claude Bailly, Neil McKerrow, Helen L. Malherbe

**Affiliations:** 1 School of Clinical Medicine, Nelson R Mandela School of Medicine, College of Health Sciences, University of KwaZulu-Natal, Durban 4000, South Africa; 2 School of Computer Science and Applied Mathematics, University of the Witwatersrand, Johannesburg 2000, South Africa; byronjgomes@gmail.com; 3 Division of Human Genetics, School of Pathology, Faculty of Health Sciences, University of the Witwatersrand, Johannesburg 2000, South Africa; arnold.christianson@gmail.com; 4 Medical Genetics, Division of Molecular Biology and Human Genetics, Faculty of Medicine and Health Sciences, Stellenbosch University Tygerberg Hospital, Cape Town 7505, South Africa; claudebailly@sun.ac.za; 5 Department of Paediatrics and Child Health, Faculty of Health Sciences, University of Cape Town, Cape Town 7935, South Africa; neilhm56@gmail.com; 6 Department of Paediatrics and Child Health, School of Clinical Medicine, Nelson R Mandela School of Medicine, College of Health Sciences, University of KwaZulu-Natal, Durban 4000, South Africa; 7 Centre for Human Metabolomics, Potchefstroom Campus, North-West University, Potchefstroom 2531, South Africa; helen@hmconsult.co.za; 8 Rare Diseases South Africa NPC, Johannesburg 2000, South Africa

**Keywords:** genetic services, medical geneticists, genetic counsellors, human capacity, human resource planning, birth defects, congenital disorders, South Africa

## Abstract

Community genetic services were introduced in South Africa almost seven decades ago, with medical geneticists and genetic counsellors being formally recognized for the past 30 years. Initial training platforms were established at academic centres countrywide, and posts for relevant healthcare professionals, including medical geneticists and genetic counsellors were created in the public sector. Despite these early advances, the number of these specialists required to address the rising burden of congenital disorders in the country remains far below required targets established by the National Department of Health. The aim of this study was to analyse the retrospective, current and projected number of medical geneticists and genetic counsellors in South Africa. The results indicate the number of practicing medical geneticists (n = 13) and genetic counsellors (n = 28) are currently at 10% and 5% of capacity targets, respectively. There is unequal distribution of these specialists between the public and private healthcare sectors, and geographical maldistribution. An alarming trend of emigration is particularly prevalent among newly qualified genetic counsellors. With the proportion of congenital disorders expected to continue to rise in coming years, together with the increasing proportion of ageing South Africans, it is imperative that health workforce planning addresses the ever-widening gap between the supply, demand and unmet need for these crucial specialists in South Africa.

## 1. Introduction

Community genetic services are healthcare measures to address the genetic health needs of a population. These measures include the prevention, detection, and care of conditions present from birth –of genetic and inherited conditions as well as those acquired post-conception during pregnancy, labour and delivery. The need for this discipline was first highlighted in 1992, as an adaptation of “*community genetics*” as coined in the 1980s [1,2,3,4]. Global efforts to define these services emerged in subsequent decades and was supported by relevant World Health Organization (WHO) Resolutions, the 2015 Millenium Development Goals (MDG) and the current 2030 Sustainable Development Goals (SDG) [2,4,5,6,7,8,9,10,11,12,13,14,15,16,17].

The aim of community genetic services is to “reduce suffering by offering care to those affected by congenital disorders and to improve health by preventing these conditions” and to ensure that those with, or at risk of these conditions, “can live and reproduce as normally as possible” [4,9,18,19,20]. Congenital disorders (CD), also known as birth defects, are defined as “any potential pathological condition arising before birth, including all disorders caused by environmental, genetic and unknown factors, whether they are evident at birth or become manifest later in life” [10,11,19,20].

Congenital disorders are a heterogeneous group of conditions that represent a significant burden of disease [20,21]. The prevention and care of these conditions requires multidisciplinary services that should be integrated at all life stages, across all levels of healthcare [11,18,21]. Implementing these services requires specialist cadres of healthcare professionals (HCP), including medical geneticists (MG), genetic counsellors (GC), genetic nurses, and genetic laboratory/medical scientists. While these genetic specialists are in short supply the world over—they are even more lacking in low- and middle-income countries (LMIC), including in South Africa (SA) [22,23].

### 1.1. Overview of South Africa

South Africa (SA) is an upper middle-income country [24]. The population of 61 million is one of the most culturally and ethnically diverse globally, as a result of historic migration from Africa, Asia, and Europe [25]. Spread across nine provinces, SA covers an area totalling 1.2 million km^2^ at the southern tip of Africa [25]. With one of the lowest fertility rates in Africa (2.34 births per woman), around a million births are recorded annually [26]. Data for 2022 indicates that 12% (n = 7.7 m) of the population were living with HIV, with the highest number of people enrolled in HIV antiretroviral treatment (ART) globally [27,28]. Increased mortality from the HIV/AIDS epidemic prior to widescale ART rollout has resulted in a younger population demographic today, with a fifth of the population aged ≤15 years and only 9% ≥60 years [25]. In 2020, neonatal, infant and under-5 mortality rates were reported as 12, 21 and 28 deaths per 1000 live births respectively, with a life expectancy at birth of 64.2 for females and 59.2 for males [25,26,29].

South Africa currently implements a dual healthcare system, with 84% of the population accessing public- and 16% private insurance-based healthcare, including a small proportion paying for private services on an *ad hoc*, out of pocket basis [30]. The private healthcare sector receives a majority share of financial and human capital, to the detriment of the public healthcare sector. This makes the South African healthcare system one of the most inequitable globally. In May 2024, the National Health Insurance (NHI) Bill was passed into law [31], aiming to redress imbalances and as a first step towards universal healthcare (UHC). Many aspects of NHI remain unresolved and contentious, and widespread implementation of NHI may take decades.

### 1.2. History of Genetic Services in South Africa

Community genetic services were first established in SA in the 1950’s with the formation of cytogenetics laboratories within publicly provided healthcare [32]. Locally referred to as genetic services, these grew into rudimentary services with two academic chairs of Human Genetics established in Cape Town (1972) and Johannesburg (1974), augmented by charity-led diagnostic laboratories in four other major centres [32,33]. By the late 1970s, genetic services were declared an “*integral part of the healthcare system funded by the state*” [34]. Development of these services is reported in detail elsewhere [13,35,36,37,38].

Despite the subsequent expansion of genetic services, creation of MG/GC posts and the development of comprehensive policy guidelines in SA [39,40], the HIV/AIDS and TB epidemics of the 1990’s reversed positive epidemiological progress initially achieved [41]. The health system now faces concurrent burdens of communicable and non-communicable diseases (NCD) due to the non-classical epidemiological transition experienced. Limited public resources are predominantly allocated to more easily diagnosed and treated communicable diseases with their perceived higher burden [41]. While implemented health interventions (e.g., Expanded Programme of Immunisation, Prevention of Mother-To Child Transmission, ART etc.) have achieved substantial reductions in child mortality, further significant reductions may only be achieved by addressing CDs as a priority health issue through comprehensive genetic services [27,42].

### 1.3. Current Genetic Services in South Africa

Today, publicly provided genetic services are largely a function of the National Department of Health (NDOH), coordinated by provincial Departments of Health (DOH) in partnership with academic institutions. A notable exception is Gauteng Province (GP), where services have historically been implemented via the National Health Laboratory Service (NHLS) through the University of the Witwatersrand (Wits).

The framework for genetic services in SA is outlined in the 2021 Clinical Guidelines for Genetic Services that details standards of care across the life course, from community to tertiary and quaternary services [21]. Human resource capacity requirements for implementing this and other relevant policies are partially addressed in the NDOH 2030 Human Resources for Health (HRH) Strategy [43], considered the only published and NDOH-endorsed capacity targets available currently [44].

#### 1.3.1. Medical Genetics

Medical and clinical genetics is the study of genetics as it relates to human health and disease, and its application to individual patients and families with CD [45,46]. Medical geneticists (MG) are medical doctors who undergo post-graduate specialist training in the field of clinical genetics, inherited diseases and CD. Their role is to diagnose and care for individuals with CD, guiding and interpreting genetic test requests and communicating resulting risks and impacts to individuals and their families [21]. Medical genetics was initially recognised as a clinical sub-specialty in SA, and the first nine MG registered with the Health Professionals Council of South Africa (HPCSA) under a “grandfather clause” in 1999 [21,35]. A further nine qualified specialists, mostly paediatricians, registered several years later after completing a newly introduced two-year training programme, under a second “grandfather clause” [35,36].

Subsequently in 2007, medical genetics was designated as a 4-year primary specialty by the HPCSA, with the first two graduates in 2012, and two more the following year [35]. It takes a minimum of 13 years to specialise as a MG in SA, including a six-year medicine undergraduate degree, two years of internship, one year of community service, followed by four years of registrar training in the field of medical genetics. All MG training is undertaken within the public sector [44] and is currentlyoffered at three of the original five academic centres: Stellenbosch University (SU), University of Cape Town (UCT) and Wits. The University of the Free State (UFS) and University of Pretoria (UP) no longer offer MG training.

#### 1.3.2. Genetic Counselling

Genetic counsellors (GC) are registered HCPs who provide education, risk assessment and support for individuals and families at risk of, or diagnosed with, a CD [21]. Informal genetic counselling services were initially established in the 1960’s, and in 1988, SA became the first African country to set up a formal genetic counselling training programme, at Wits [35,47]. It takes a minimum of seven years to become a GC in SA. The GC training programme was established for those with a relevant undergraduate Honours degree (four years), as a two-year Master’s (MSc) in Genetic Counselling and a two-year internship, with the first year of the internship overlapping with the second year of the degree. In 1992, genetic counselling was formally recognised as a health profession by the HPCSA [48]. A similar Master’s course was subsequently established at UCT in 2004 [47].

Within this SA landscape and the context of the revised Clinical Guidelines for Genetic Services [21]—the aim of this study was to undertake a retrospective and current audit of MG/GC in the country to date, and to generate modelled prospective capacity estimates up to 2045, and evaluate these outputs against recommended capacity ratios.

## 2. Materials and Methods

### 2.1. Retrospective and Current Data Compilation

Several data sources were used to collate the audit information for MG/GC from 1995 to April 2024. The primary source of data for MG/GC qualifying in SA was the freely available, online HPCSA database (https://hpcsaonline.custhelp.com/app/i_reg_form, accessed on 27 April 2024) to extract a list of all registered MG/GC in SA. Search criteria used were “*genetic counsellor*” and “*medical genetics*” for ‘register’ and ‘category’ groupings, respectively. Extracted information was recorded using Microsoft Excel.

Since the HPCSA register does not specify details on registered HCPs i.e., if currently practicing, and if so, which sector etc., further information was required for each individual HCP of both cadres. Emails were sent to the clinical heads of the three academic genetic units (SU, UCT and Wits) requesting a summary of MG/GC currently in-training with estimated completion dates. Responses were added to the Microsoft Excel worksheet.

All registered MG/GC residing in SA were also contacted individually via email to confirm details and identify those working in the private sector. Contact details were either known to the authors, supplied by the clinical heads, or obtained via a Google search. Information requested included: confirmation of HPCSA registration year, details of current practice (if practicing), healthcare sector (public/private/both) and their contribution to each.

All HPCSA registered and non-registered MG/GC known to be no longer practicing in SA were also appropriately recorded. This was based on information known to the authors, supplied by clinical heads or via other contacts in the sector with relevant institutional memory.

Data gathered from all sources were collated and anonymised in Microsoft Excel. The employment status of every individual was categorised as privately or publicly practicing and location, deceased, retired, emigrated, working in academia/research, or not practicing (Supplementary Table S1). Descriptive statistics were used to analyse the collated retrospective and current data.

Informed consent was obtained from all clinical heads and individual MG/GC who shared information.

### 2.2. Modelled Prospective Data

The projected capacity requirements for MG/GC from 2025–2045 were generated by adapting an existing 2019 capacity model projecting the supply and need for medical specialist resources in SA by Wishnia et al. [44]. Themodel’s original Python code was shared via a Data Transfer Agreement (DTA) with Rare Diseases South Africa NPC (Dr Jodie Wishnia, Personal Communication). Details on the approach used in the original model are available in the Wishnia et al. 2019 report [44].

For the current study, the following changes were made to the model assumptions (see Table 1 and Table 2 for details):

(a) ***Scope:*** The model scope was adapted to produce outputs solely for MG/GC using the granular information compiled in this study (Table 1), and not for the 70 HCP cadres included in the original model [44].
(b) ***Time period:*** Modelled estimates were produced for 2025 to 2045 (original model 2019 to 2040).
(c) ***Population data:*** Updated South African population data sourced from Thembisa version 4.7 were used [28,48]. Since this dataset included population estimates until 2030 only it was necessary to extrapolate public healthcare population data for 2031 to 2045. Extrapolation assumed stable year-on-year population growth of 1.48%, based on the average growth rate observed in Thembisa data from 2024 to 2030 [28,48]. The portion of the population served by private healthcare remained unchanged as per the original model throughout.
(d) ***Starting population:*** 2024 was used as the starting point for populations of MG registrars and GC interns. This was split by age, sex and training study period (1st year/2nd–4th year for MG registrars and 1st year/2nd year for GC interns) based on the data compiled, as outlined above. For cases when specific MG/GC ages were unavailable, estimates were derived based on the year of qualification for MG/registrars and the year of postgraduate degree for GC/interns indicated in the HPCSA online database.
(e) ***Capacity Rates:*** For MG, the recommended capacity rate used was as per the previous model sourced from the NDOH 2030 HRH Strategy of 0.21 per 100 000 population [43]. Since no capacity ratio is indicated for GC in the HRH strategy, the previously recommended ratio of four GC per MG was implemented [39,49].
(f) ***Qualification rates:*** The model projections assume that the number of MG registrars/GC interns remains stable i.e., as they qualify, they are replaced with a new intake of registrars/interns. The qualification rates (quantifying newly qualified MG/GC entering the workforce) for MG registrars and GC interns were calculated using the compiled actual numbers of registrars and interns:
  - The number of MG registrars who qualified thus far in 2024 (n = 2) was divided by the total number of registrars in their 2nd to 4th years of training in 2023 (n = 5). The resulting ratio of 0.40 was used as the model qualification rate for MG registrars (versus 0.23 used for all specialists in the original model) [44].
  - Genetic counsellor intern training is a minimum of two years. The number of interns who graduated in 2023 (n = 1) was divided by the number of interns in their 2nd year of internship in 2023 (n = 3). This equated to a qualification rate of 0.33 for GC interns.
(g) ***Movement between healthcare sectors*:** The probability of MG moving from the public to the private sector is low, therefore the model probability remained at 10% until retirement age (60–65 years), when increased to 50% due to mandatory retirement ages in the public sector (Table 2). For GC, based on the number currently employed privately, movement between the sectors is more common, making the probability of GC moving to private practice a higher assumption in the model. The probability of moving back to the public sector from private for both cadres was aligned to the original model, which assumed that HCP may move back to the public sector closer to retirement to “give back” to the sector.
(h) ***Movement between part-time and full-time:*** Most MG/GC in SA are female, making both cadres more likely to move from full-time to part-time in their reproductive years, between ages 32–37 and then back to full-time as child-rearing responsibilities decrease (Table 2), as per the original model assumptions [44,50]. While the original model split these probabilities between males and females, a combined probability was used in the updated version. Since collated MG/GC dataset was mostly female, the model would have required extensive re-working to include a gender split and was not deemed necessary.
(i) ***Emigration:*** Using collated retrospective data, emigration probabilities were calculated separately for MG and GC, by dividing the number of emigrations to date by the total number of those registered with the HPCSA for both cadres. This total probability for each cadre was then split by age group using the estimated ages of the MG/CG at the time of emigration (Table 2).
(j) ***Scenarios Modelled:*** As per the original model, four different scenarios were prospectively modelled:
  - ***Scenario 1:*** Current capacity rates for MG/GC 2024 (using compiled data) were used to project capacity for the *public* healthcare population for 2025–2045.
  - ***Scenario 2:*** Current capacity rates for MG/GC 2024 (using compiled data) were used to project capacity for the *private* healthcare population for 2025–2045.
  - ***Scenario 3*:** The recommended rate of 0.21 per 100,000 population (NDOH 2030 HRH Strategy [43]) was used to generate capacity projections for the total healthcare population for 2025–2045. For GC the rate of four GC for every one MG was used, as per previous NDOH recommendations [39,49].
  - ***Scenario 4:*** The required rates to reach the recommended target ratios in 2045 (Scenario 3) were calculated using linear interpolation. The required rates (i.e., how many MG/GC should be annually added to the system to meet the 2045 target) were computed for each year from 2024–2025. This method assumes linear annual increments in the required ratio. This differed from the original Wishnia model Scenario 4, which enabled the capacity rate to change and respond to evolving capacity need caused by changes in the burden of disease over time [44]. While this approach worked well for HCP cadres allocated to a sub-population (e.g., paediatric population), it was not deemed relevant for MG/GC since both these cadres serve the total population and are known to be extremely scarce. Rather, quantifying the gap between current and recommended capacity by 2045 was deemed to be more appropriate for this study.

**Table 1:** Supply side assumptions for the original model [44] and the adapted model in the current study for medical geneticists (MG) and genetic counsellors (GC).

Assumption	Original Model * [44,51]	Updated Model (Current Study)
Age range of MG registrars	28–50 years	
Age range of MG	31–75 years	
Age range of GC	-	26–75 years **
Geography	South Africa	
Remunerated work outside public sector	70% of time public, 30% private	MG: 95% of time public, 5% private ** GC: 87.5% of time public, 12.5% private ** Average of the two rates combined used 91% public, 9% in private
**Transition probabilities**		
Retirement age range—public	55–65 years	
Retirement age range—private	65–75 years	
Death—Female	25% of SA85–90 Light	SA85–90 Light Male & female rates weighted for average MG/GC calculated on retrospective data **
Death—Male	45% of SA85–90 Light	
Move: public to private	Range of probabilities between end of registrar-ship and retirement	Range of probabilities between end of registrar-ship and retirement **
Move: private to public	Range of probabilities between 45–65 years	Range of probabilities between 45–65 years **
Move: full-time to part-time (female)	A range of options between 32–39 years	Male/female data aggregated. Range of options for MG/GC ***
Move: full-time to part-time (male)	A range of options between 50–65 years	Male/female data aggregated. Range of options included for MG/GC ***
Move: part-time to full-time (female)	Range of possibilities between 40–55 years	Range of possibilities, split between MG/GC, between 40–55 years **
Move: part-time to full-time (male)	0 (move at end of career)	Original model assumptions
Probability of emigrating (medical geneticists)	Range of possibilities between 31–45 years, all specialties.	Range of possibilities between 31–50 years **
Probability of emigrating (genetic counsellors)		A range of possibilities between 25–40 years **
Full-time equivalent (FTE) calculations for registrars	1st year registrar 60% FTE 2nd–6th year registrars weighted to derive weighted average 80% FTE	Original model assumptions: 1st years 60% FTE 2nd–4th years 80% FTE
Full-time equivalent calculations for part-time cadre	Guided by public sector 5/8ths posts (62.5%)	N/A All MG/GC posts are FT

* Assumptions used in the original model by Wishnia et al. [44]. ** Adapted assumptions based on trends identified in retrospective data compiled for this study. *** The original model [44] split certain data points between males/females. For the current study, since the majority of MG/GC were female, the use of male/female in the model were replaced/renamed as MG and GC to avoid model rebuilding.

**Table 2:** Detailed probability assumptions used in the original model versus the updated model for medical geneticists (MG) and genetic counsellors (GC).

Probability Moving Public to Private				
Age Range	Original [45]		Updated: Medical Geneticists	Updated: Genetic Counsellors
31–35 years	25%		10%	50%
35–40 years	25%		10%	30%
40–50 years	15%		10%	10%
50–55 years	25%		10%	30%
55–60 years	25%		10%	30%
60–65 years	35%		50%	40%
65–75 years	0%		0%	0%
**Probability Moving Private to Public**				
Age Range	Original [45]		Updated: Medical Geneticists	Updated: Genetic Counsellors
40–50 years	10%		20%	20%
50–60 years	5%		5%	5%
60–65 years	2%		2%	2%
**Probability Moving Full-Time to Part-Time**				
	Original Model		Updated Model	
Age Range	Female	Male	Medical Geneticists	Genetic Counsellors
32–35 years	10%	0	5%	15%
35–37 years	25%	0	20%	25%
37–39 years	15%	0	15%	15%
50–55 years	0	15%	10%	10%
55–60 years	0	20%	10%	10%
60–65 years	0	25%	25%	25%
**Probability Moving Part-Time to Full-Time (Females Only)**				
Age Range	Original		Updated: Medical Geneticists	Updated: Genetic Counsellors
40–45 years	50%		30%	30%
45–50 years	35%		50%	50%
50–55 years	25%		25%	25%
**Probability of Emigration**				
Age Range	Original		Medical Geneticists	Genetic Counsellors
25–30	0		0	22%
31–35	4%		2%	2%
36–40	6%		12%	5%
41–45	3%		12%	0
46–50	0		7%	0

Once the coding assumptions were updated as detailed, the model was regenerated in Python in July 2024. The model outputs were exported and pasted into Microsoft Excel (Supplementary Table S1), for analysis.

Descriptive statistics were used to evaluate and compare the four different scenarios. To enable international comparability, all rates used were per 100,000 total population.

The study was conducted in accordance with the Declaration of Helsinki and approved by the Institutional Ethics Committee of the University of KwaZulu-Natal, South Africa (protocol code BREC/00006584/202; 26 April 2024) for studies involving humans.

## 3. Results

### 3.1. Retrospective Data

A total of 41 MG and 54 GC were included in the study. Of the 41 MG, 38 were identified from the HPCSA register and three from other sources. All of the 54 GC included were identified from the HPCSA register. An additional three individuals were listed under “Genetic Counsellor” on the HPCSA but were excluded as one is a MG and two are medical scientists. A total of 16 MG and 33 GC, including the clinical heads of department, were contacted via email. The majority of MG (15/16, 94%) and GC (33/34, 97%) contacted responded to the audit information request. All clinical heads of department responded. Full anonymised data is included in Supplementary Table S1.

#### 3.1.1. Geographical Distribution

The geographical distribution of MG/GC retrospective data was aligned with the original academic centres of UCT, US, Wits, UP and UFS, based in the Free State (FS), GP and the Western Cape (WC).

#### 3.1.2. Medical Geneticists

Of the 41 MG qualified/registered between 1 January 1997 and 30 April 2024, only a third (13/41) are currently practicing in SA (32%), including nine (69%) in the public and four (31%) in the private sector. The majority of practicing MG are female (10/13, 77%).

**Figure 1:**
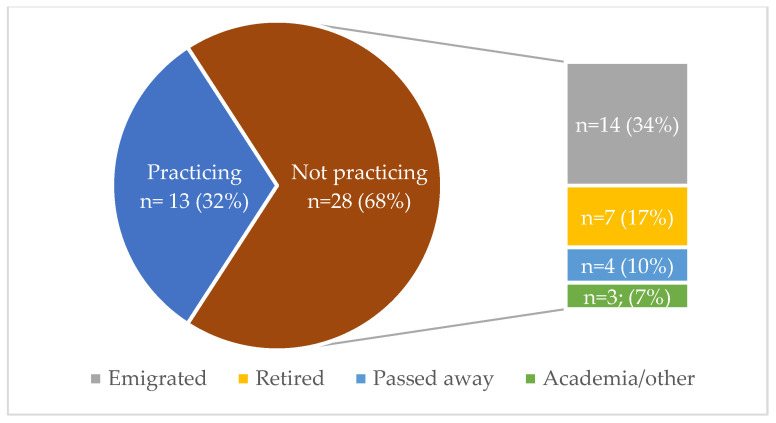
Status of medical geneticists qualified/registered in South Africa since 1997, with reasons for not practicing. Note: Academic/other indicates those who remain HPCSA registered but are working in other sectors/not practicing.

**Figure 2:**
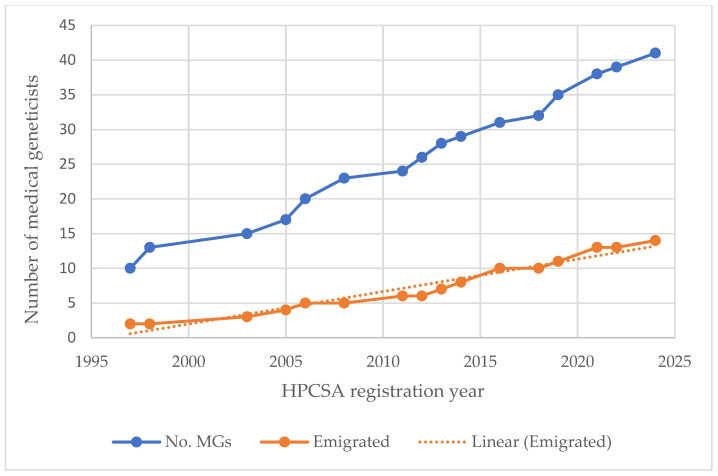
Cumulative number of medical geneticists who have registered in SA and emigrated (1997 to April 2024).

Of the 28 MG no longer practicing (Figure 1), half emigrated (14/28, 50%), a quarter (7/28, 25%) retired, and 4 (14%) are deceased. There is an upward trend of MG leaving the country aged 36–45 years (Figure 2). Of the 14 emigrations, seven (50%) qualified/registered since 2014. Of the two most recently qualified MG at Wits (2024), neither are practicing in SA—one emigrated and the other cannot practice in the public sector as there is no post available.

#### 3.1.3. Genetic Counsellors

A total of 54 GC registered with the HPCSA from 1995 to April 2024, of which 26 of (48%) are not practicing in SA. Just over half (28/54, 52%) are currently practicing in SA, of which 18 are in private (64%) and ten in the public sector (36%). Of those currently practicing, 96% (27/28) are female.

**Figure 3:**
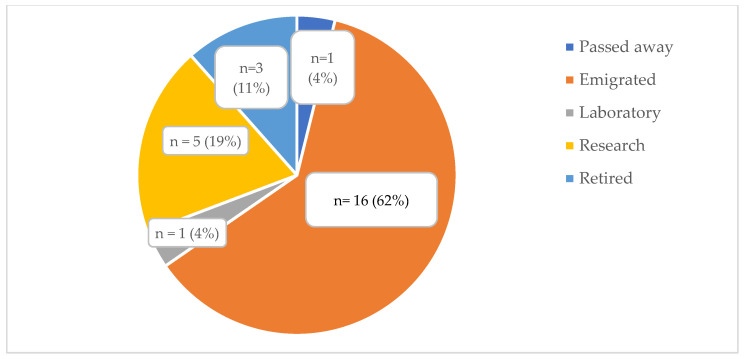
Breakdown of reasons for non-practicing genetic counsellors qualified/registered in South Africa since 1995.

Of currently non-practicing GC in SA (26/54, 48%), five moved into research positions, three retired, one is deceased, and one works as a genetic specialist in a private laboratory (Figure 3). The majority (16/26, 62%) of registered but non-SA practicing GC have emigrated, and of these, 44% (7/16) are aged between 26–30 years, with the majority (11/16; 69%) qualifying/registering in the past ten years (2016 to 2024) (Table 3).

**Figure 4:**
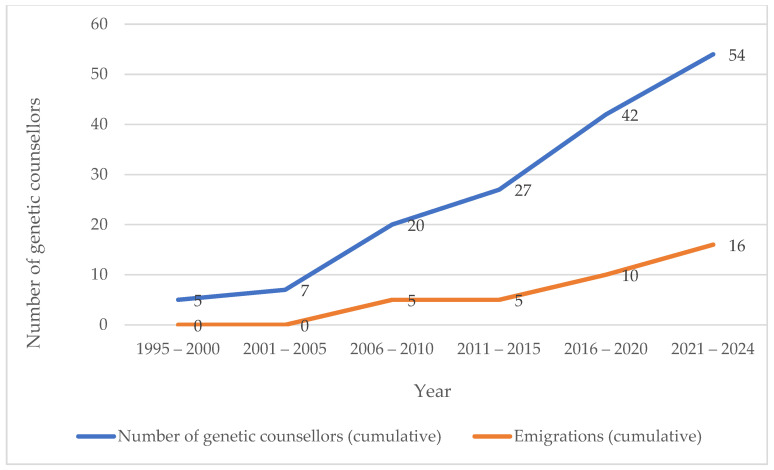
Cumulative number of genetic counsellors who have registered and emigrated from 1995 to April 2024.

The overall average age of emigrating GC was 29.6 years of age (Figure 4), but this has decreased to 27 years of age in the past ten years (2014–2024), indicating an increase in immediate post-qualification emigration.

### 3.2. Current Data

#### 3.2.1. Geographical Distribution

**Figure 5:**
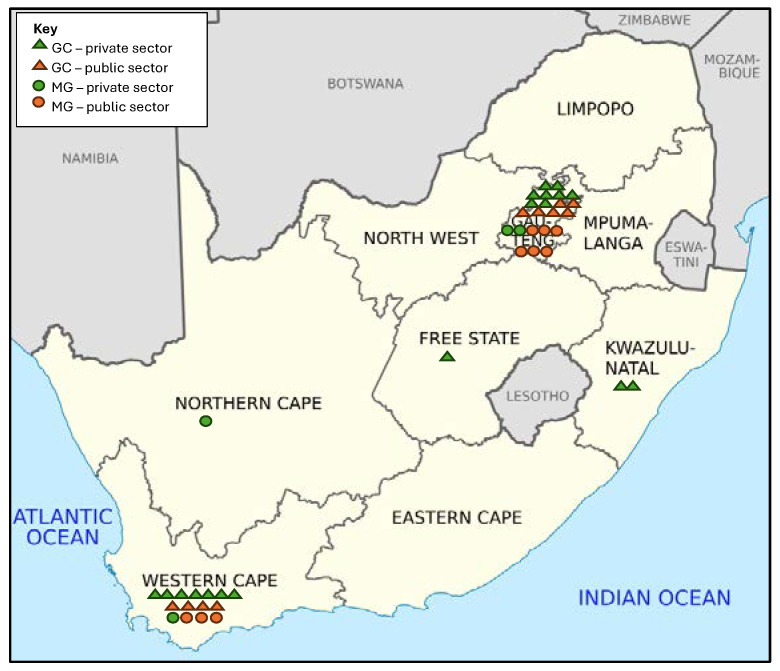
Population and numbers of practicing medical geneticists (MG) and genetic counsellors (GC) by province in SA across both public and private healthcare sectors. (image: https://www.statista.com/statistics/1112169/total-population-of-south-africa-by-province/, accessed on 30 May 2024).

Table 3 and Figure 5 show the geographical distribution of currently practicing MG/GC across SA. Five of the nine provinces have no GC and six have no MG. Publicly practicing MG are based in GP (6/9, 67%) and WC (3/9, 33%) only. Privately practicing MG (100% private) are in GP (2/4), WC (1/4) and Northern Cape (NC) (1/4).

For GC, those practicing privately are in GP (8/18), WC (7/18), KwaZulu-Natal (KZN) (2/18) and FS (1/18). Publicly practicing GC are located in GP and WC only, operating at 5% and 9% provincial capacity respectively, leaving seven provinces with no publicly practicing GC. Privately practicing GC are found in five provinces, supplementing public GC in GP and WC. A remaining four provinces have no practicing GC in either sector.

#### 3.2.2. Public/Private Sectors

The total number of MG/GC currently practicing in 2024 across both sectors (Scenarios 1 and 2) was compared against recommended HRH rate outputs for Scenario 3 (Table 4). Currently, 13 MG and 28 GC practice in SA across both healthcare sectors, indicating combined capacity of 6%. Capacity levels vary for MG/GC between healthcare sectors, with the public sector operating at 3% capacity, (8% of required MG, 2% of GC), versus the private sector operating at 24% capacity (22% of required MG and 24% of GC). Most MG practice in the public sector (9/13, 69%), and most GC in the private sector (18/28, 64%).

Of the 13 practicing MG, 38% (n = 5) are aged 41–45; almost a quarter (3/13, 23%) are >60; and 15% (2/13) are 56–60 years (Table 4). All MG practicing in public sector are aged >40 years, and a third of these (33%, 3/9) are ≥60 years of age. Six of the nine MG (67%) in public spend a small proportion of time (5% on average) consulting in the private sector.

The majority (70%) of GC practicing in the public sector are <36 years of age (7/10). Public sector GC spend an average of 7% of time consulting privately. Two privately practicing GC also spend a portion of their time, 40% and 10%, in the public sector.

#### 3.2.3. Ongoing Training

Of the nine MG registrars in-training, two are supernumerary registrars (one each at Wits and UCT) (Table 5), who are funded externally, usually by their home country. Supernumerary registrars are required to return to their home country upon completion and are not permitted to practise in SA following qualification—unless relevant visa or immigration processes are undertaken and posts are available that cannot be filled by qualified South African citizens [52].

There are currently eight GC interns and eight GC students, in-training at UCT and Wits (none at SU). Due to curriculum revision, there was no new intake of GC students at UCT in 2024. Wits currently has five students and two interns but will have no new intake in 2025 due to planned curriculum revision.

### 3.3. Modelled Projected Data

Projected outputs from the revised model are divided into the four scenarios:

- ***Scenario 1:*** Current rates for public healthcare sector in 2024 projected 2025–2045.
- ***Scenario 2:*** Current rates for private healthcare sector in 2024 projected 2025–2045.
- ***Scenario 3:*** Recommended 2030 HRH rates projected 2025–2045.
- ***Scenario 4:*** Required rates from 2025 to reach Scenario 3 ratio target in 2045.

**Figure 6:**
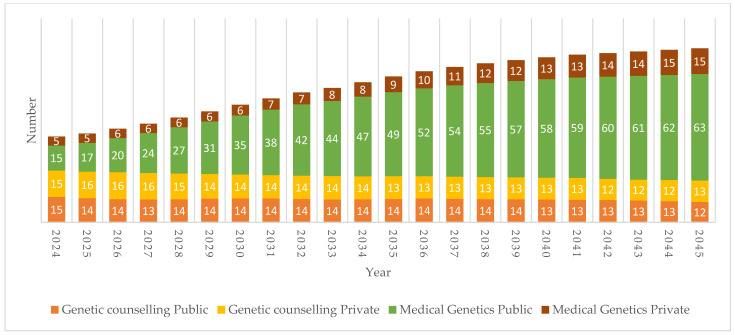
Projected growth from 2024 to 2045 in the public and private healthcare sectors for medical geneticists and genetic counsellors based on current actual (2024) rates and including medical genetics registrars and genetic counselling interns (Scenarios 1 & 2).

The results indicate that based on current (2024) capacity rates and the model assumptions, MG numbers will increase from 18 to 77 across both healthcare sectors by 2045, while combined GC numbers will decrease from 31 to 27 over the same 20-year period (Figure 6). This incorporates ongoing additions and losses to the two cadre populations through training, losses to emigration, retirement, death, exiting the sector, movement between full-time/part-time, and movement between public/private. In the public sector, the number of MG (49/59, 83%) is expected to grow more than privately practicing MG numbers (10/59, 17%), while GC numbers will remain stagnant, with a slight decrease in both sectors by 2045. All projected rates for GC remain far below the HRH recommended rates throughout the 20-year period [43].

#### Analysis of Target Ratios across Scenarios

**Figure 7:**
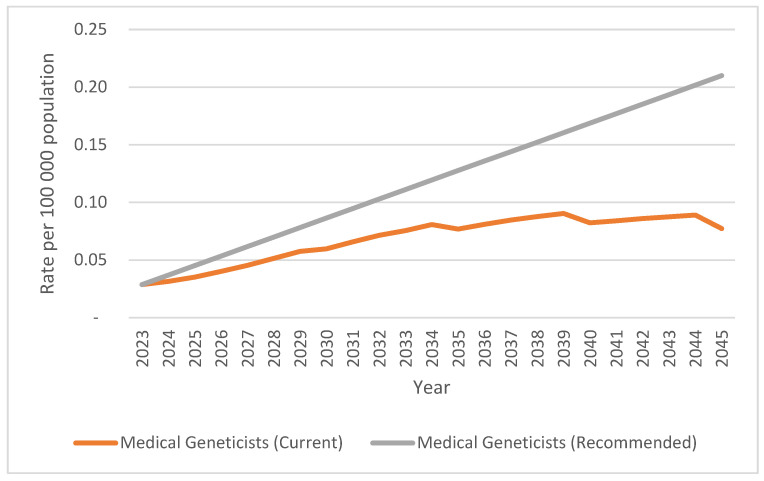
Comparison of current capacity (2023) of medical geneticists (MG) projected 2024–2045 (Scenario 1 & 2) with HRH recommended target ratio of 0.21 MG per 100 000 population projected by 2045, including MG registrars in training (Scenario 3) [43].

**Figure 8:**
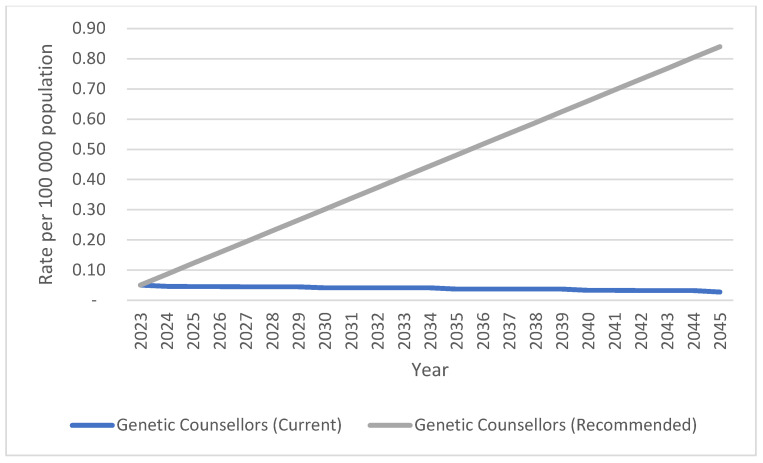
Comparison of current capacity (2023) of genetic counsellors (GC) projected 2024–2045 (Scenario 1 & 2) with HRH recommended target of 0.84 GC per 100,000 population projected by 2045, including GC interns in training (Scenario 3) [39,49].

**Figure 9:**
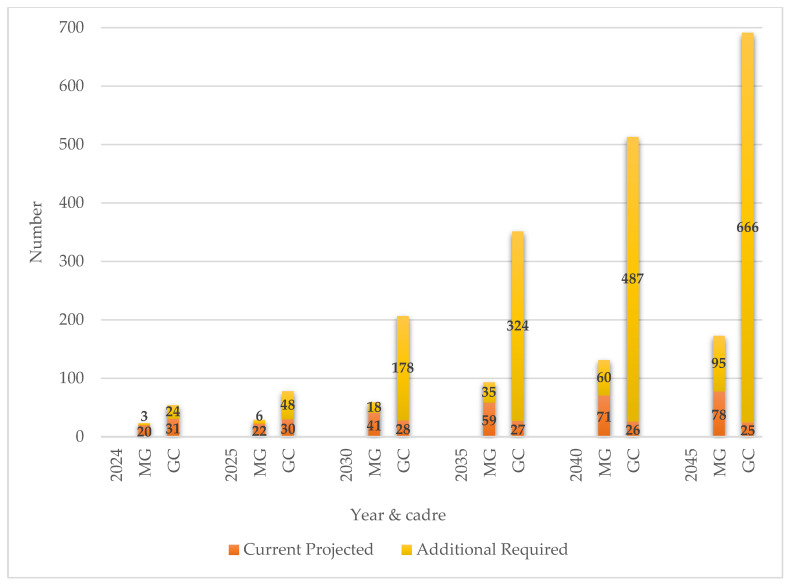
Current projected and additional required number of medical geneticists (MG) and genetic counsellors (GC) from 2024 to 2045 in five-year intervals. Data labels indicate the additional number of MG/GC required at each point in time, in addition to projected numbers, to reach recommended capacity by 2045.

Figure 7 and Figure 8, show the deficit between the current and recommended HRH capacity rates (Scenario 3) for both MG and GC, respectively. In Figure 9 the required rates and numbers of MG/GC are quantified, to reach the recommended capacity rates of 0.21 and 0.82 for MG and GC respectively by 2045.

Figure 7 highlights the current and ongoing shortfall in the capacity rates of MG in comparison to those recommended from 2024–2045. While the capacity rate is predicted to double if current capacity rates continue, it still falls far short of the required rate of 0.21 per 100,000. Similarly, Figure 8 compares the same for GC—and indicates a decline in the capacity rate of GC by 2045.

While current MG/GC are far below the recommended numbers, Figure 9 shows a potential progressive approach to reach the recommended capacity targets by 2045 (Scenario 4). The number of additional MG/GC that need to be trained, over and above existing capacity, are quantified. For example, by 2045, with a predicted SA population of 82.263 million, a further 95 MG are required in addition to the 78 projected, to reach the target ratio of 0.21 MG per 100,000 population (equating to 173 MG). Similarly, for GC, in addition to the 25 projected, an additional 666 must be trained to reach the target ratio of 0.84 per 100,000 (equating to 691 GC for the predicted population of 82.263 million).

## 4. Discussion

The aim of this article was to collate and analyse retrospective, current and modelled prospective capacity ratios of MG/GC in SA against recommended ratios. While 41 MG and 54 GC have qualified/registered with the HPCSA since 1997 and 1995 respectively, only 13 MG and 28 GC remain practicing in SA today. These numbers are far below HRH recommendations and the rates required to meet these recommended targets by 2045 [43]. Practicing MG/GC are unevenly distributed across the country, resulting in inequity both between the public/private sectors and geographically between provinces.

In SA in 2012, genetic services met 10% of the population need [35,47]. Little has changed in the subsequent decade, with continued population growth, growing CD health need, and scarcer resources—despite numerous studies and advocacy efforts highlighting this capacity shortage [22,35,36,37,38,41,53].

### 4.1. Medical Geneticists

The 2030 HRH strategy recommends a countrywide target ratio for MG of 0.21 per 100,000 population equating to one per 476,000 people, equating to 132 MG recommended countrywide in 2024—versus the 13 currently practicing which indicates only 10% capacity overall [43,44]. Current MG levels in public (8%, n = 9) and private healthcare (22%, n = 4), equate to 1 MG per 4.7 million total population, including 1 per 5.7 million in public and 1 per 2.4 million in private [43]. These capacity levels are insufficient to serve the population in either sector.

There is also geographical maldistribution of MG across the nine provinces, with seven of the nine provinces, representing 61.7% of the SA population, without an MG. As the second most populous province in the country (12 million; 19% total population) KZN has no MG practicing in either healthcare sector, and only limited support from two paediatricians with an interest in genetics. Similarly in Pretoria, one paediatrician (genetics) offers paediatric genetic services—but is due to retire in the next five years with no succession plan in place.

#### 4.1.1. Ageing Workforce

The age range of practicing MGs is also concerning. In the public sector, a third are ≥60 years of age and close to retirement (mandatory at 65), although some may practice privately post-retirement. Of the remaining six in public, only two are between 46–59 years of age, leaving a large gap between the older, experienced MG and those newly qualified. The uptrend in emigration of more recently qualified MG is also alarming but follows a similar pattern for other clinical specialists [54]. The loss of experienced MG to the country may have a lasting effect on the training potential of future generations.

#### 4.1.2. Private Practice Challenges

When MG first became a designated HCP category, they were solely employed in the public sector (tertiary hospitals) [35]. Today, four MG practice exclusively in the private sector in GP, NC and WC. Few MG choose to go into private practice due to the significant workload and challenges related to medical insurance billing codes—which are not discipline specific. This results in reimbursement not accurately reflecting the time/expertise associated with a consultation. The relatively high cost of genetic testing, often not covered by medical insurance, also makes private practice difficult to sustain (Dr Claude Bailly, personal communication). Addressing these issues could result in expanded MG capacity in the private sector and help retain more MG in SA, vital for future NHI implementation.

#### 4.1.3. Lack of Public Posts

The projected number of MG is expected to increase by 2045, particularly in the public sector. This is partly due to the number of qualifying registrars used in the model assumptions, while considering losses to the system. However, there is a disconnect between the training of MG registrars and public service posts for MG. The number of registrars in-training is not equivalent to the number of available posts for MG—preventing newly qualifying MG from practicing in the public sector in SA.

The lack of posts for MG has been exacerbated by a nationwide, ongoing moratorium on new post creation. Historically, implementation of this moratorium has varied between provinces, with some able to fill current posts only and others, e.g., KZN, able to create new posts since 2016 with approval from KZN provincial treasury. However, in August 2023, SA National Treasury issued a letter to all government departments, including the National and Provincial Departments of Health DOH, outlining new austerity measures including the further freezing of posts [55]. Comprehensive health workforce planning is essential, particularly in the context of NHI, to ensure that these supply gaps are minimised [51].

##### Burnout

While empiric data is lacking in SA, it is estimated that a full-time MG working in the state healthcare sees an average of between 15–30 patients per week, 48 weeks per year, averaging 1000 patients annually—in addition to non-clinical work including teaching, research, administration and marketing (Dr Claude Bailly and Dr Mike Urban, personal communications). The number of patients seen by a MG per week depends on the unit and amount of support staff and HCP—including the number of MG consultants, MG registrars and genetic counsellors. Those in-post are already working at full-capacity, with clinics booked months in advance and many MG logging additional, unreimbursed overtime. Unsurprisingly, this excessive pressure is causing many MG to burnout, which was further exacerbated during the recent COVID-19 pandemic, as for other HCP. Targeted interventions are required to mitigate this job-related stress and burnout [56].

### 4.2. Genetic Counsellors

The 2030 HRH strategy [43] does not include capacity recommendations for GC (only for psychologists/vocational counsellors). Previous NDOH recommendations indicated there should be four GC for every one MG, (0.84 GC per 100,000 population) [21,49]. The 28 GC currently practicing in SA equates to only 5% (28/529) capacity across both sectors; 2% capacity in public (10/455) and 24% (18/74) in private. Even if these requirements are halved, i.e., two GC per MG, with 264 GC required countrywide—current numbers would equate to only 10% GC capacity. GC currently practicing in public are exclusively located in GP, (5% capacity), and WC (9% capacity). The 19 privately practicing GC are based in four provinces, including FS, which has no GC in the public sector, and no MG in either sector.

The number of GC in both sectors is projected to decrease by 2045, exacerbating the current gap as well as the geographical maldistribution of GC services in SA. Without effective planning and addressing specific challenges facing this HCP cadre outlined below, SA may very well see a collapse of GC services in the public sector in the coming decades.

#### 4.2.1. Lack of Appropriate and Standardised Renumeration

GC posts in SA are not included in the Occupation Specific Dispensations (OSD) and resulting GC salaries offered are based on non-professional scales and are not comparable to other HPCSA registered Masters professionals such as psychologists. Salaries for GC also vary between provinces. Those provinces offering higher salaries are more attractive to qualified GC. Resolving the issue of appropriate, standardised reimbursement scales for GC would help clarify the GC capacity requirement in the country.

#### 4.2.2. Lack of Public Posts

Initial gains in training and post creation in the public sector have all but ceased. In GP, eight permanent posts established via the NHLS in the 2000’s has decreased to five [36] and no support is provided by the Gauteng DOH to support genetic services in the province. There are currently only four full-time posts in the Western Cape [38]. However, this is not a new challenge—between 2011 and 2013 GC training was placed on hold due to the lack of employment opportunities following graduation [38].

The lack of GC public posts available in SA has caused a major shift in capacity from the public to the private sector, with almost double the number of GC practicing in private healthcare (serving 16% of population) versus the public sector that serves 84% of the population [57]. Private practice also offers greater flexibility in hours and focuses solely on counselling, versus the mandatory full-time posts in public which involve additional teaching, administration and other roles [44].

#### 4.2.3. Emigration

The data compiled in this study shows that in the last ten years, 70% of GC graduates have emigrated, with the age of emigration decreasing to immediately following qualification. The lack of local posts, combined with other societal pressures are thought to contribute to these high rates of emigration.

In essence, SA is training inadequate numbers of GC to serve the population, and the majority of the few trained GC are being lost through emigration for the benefit of other, mainly high-income countries that value their skills—to the detriment of the SA population. Those GC remaining in SA face similar pressure and burnout to MG. This begs the question: *Is it ethical to continue GC training when there are no posts available in SA and no plans in place to address the deficit? What are the ethics of not allocating and funding posts for GC to address the genetic health of the population?*

### 4.3. Limited Training Capacity

There are clearly challenges in the interface between the training platforms of both MG/GC and the resulting public service. This has compromised both the retrospective and current capacity of these two HCP cadres and threatens their future supply.

#### 4.3.1. Medical Geneticists

While the early “grandfather clauses” enabled the addition of 18 MG to the public healthcare system over a short period in the 1990s/2000s, subsequent training of MG and the number of available posts following qualification is inadequate. The reduction of the initial five MG training centres to three further slowed the replacement of MG lost to the system and has led to a heavily skewed distribution of MG in only the two provinces with training centres (WC and GP). A previously designated genetic unit at UFS remains non-operational following the Director’s retirement and relocation of the (now qualified) registrar to NC in 2019/2020—forcing referrals to other provinces. At UP, a previously established medical genetics department closed in 2001 [35].

The number of registrars that can be trained at each of the three remaining training centres (SU, UCT, Wits) [58] is limited. At Wits, there are currently eight registrar training posts equating to 1.6 registrars per medical geneticist. The biggest obstacle to the number of MG registrars that can be trained is the availability of in-post MG to supervise and provide adequate training (Prof Amanda Krause, personal communication). Both UCT and SU have the capacity to train four MG registrars each but have only one funded post available at each centre, despite unsuccessful motivation for additional posts (Dr Karen Fieggen, personal communication). The fewer MG permanent posts and registrar funded posts available, the fewer registrars that can be trained.

Of the nine MG registrars in training in GP and WC due to qualify between 2024–2029, two are supernumerary registrars—foreign students who return to their home country upon completion of their training. This leaves the potential future increase in MG in SA at only seven. However, the lack of available public MG posts may leave these and future registrars unable to practice after qualifying, as experienced by the two most recently qualified—causing one to emigrate and the other with no prospects.

#### 4.3.2. Genetic Counsellors

Wits and UCT are the only two GC training centres in SA, with SU offering GC internships only [35]. The number of GC students and internships is limited to the number of practicing GC and the number of paid internships available at these institutions. The HPCSA recommended ratio of interns to qualified GC is 4:1, however, in practice this ratio depends on the size of the training centre determined at accreditation. Only HPCSA registered GC practicing more than three years can supervise interns (Katryn Fourie, personal communication). Collectively, the lack of paid internships, insufficient experienced GC in-post (two thirds of those currently employed are ≤40 years of age) and limited training centres are severely curtailing GC training capability.

In the long term, the compromised training platforms for both MG and CG will inevitably affect the future supply of these specialists in the country [51].

### 4.4. Additional Challenges

Other key challenges identified in this study include:

#### 4.4.1. Growing Genetic Health Need

While SA currently has a young population demographic, the proportion of older people is rapidly increasing and by 2050, it is expected that 15.4% of the population will be aged ≥60 years by 2050, compared to only 9% today [59,60]. As SA transitions epidemiologically [41,59], the proportional contribution of CDs to child mortality will continue to increase together with this ageing population, following the trend of HIC and emerging global studies [61,62,63]. On the current trajectory, neither MG or GC numbers will reach recommended levels by 2045, leaving the genetic health of the population largely unaddressed and at risk in the coming decades.

#### 4.4.2. Insufficient Planning and Implementation

While multiple health strategies have been published by NDOH in recent years [21,43,64,65], these lack detailed, costed implementation plans [66]. There is also a disconnect between the NDOH producing relevant policies and plans but implementation being undertaken by the Provincial DOH. As a result, implementation is fragmented and inadequate, further contributing to the lack of posts and funding for relevant HCP, with negative consequences for patients and their families. Clarity is also needed countrywide on whether genetic services should fall under NDOH, Provincial DOH or NHLS jurisdiction, both in the current setting and future NHI implementation [36].

#### 4.4.3. Inadequate Clinical and Administrative Support

The extensive network of genetic nurses appointed in the 1970s filled the gap in genetic services up until the HIV/AIDS epidemic, when they were re-assigned to primary healthcare clinics to address this infectious disease [58]. Little has been done to replenish these nurses and few remain or have been newly trained—despite their vital role in seeing patients and referring more complex cases to GC/MG [58], to reduce the tertiary level workload. Within the hub and spoke model of genetic services outlined in the Clinical Guidelines for Genetic Services [21], genetic nurses are considered essential across all provinces, particularly in the context of NHI. The NHI Green and White Papers both clearly delineate the central role of nurses in the multidisciplinary team-based approach to public healthcare [51].

#### 4.4.4. Under-Utilisation of Telemedicine and Outreach

While telemedicine is a viable option, as demonstrated during the COVID-19 pandemic [67], this rarely occurs in the public sector since patients in rural populations may lack the IT infrastructure, data and skills required. In addition, in the public sector most MG/CG are working at full capacity as they are not only involved in counselling but also in research, teaching, marketing and administration duties [47].

In rural and sparsely populated areas, genetic services are non-existent, except for occasional outreach clinics which have decreased in recent years due to lack of funding and capacity [35]. As a result, many patients are referred across provincial borders placing further pressure on already limited resources.

Telemedicine may assist in filling this gap via a relatively low-cost solution, if additional HCP capacity is made available through additional post provision.

### 4.5. Study Limitations

The study was limited to two cadres of HCP, MG/GC, and excludes medical scientists and genetic nurses. Further studies are required on these other HCP cadres to highlight capacity shortages and requirements to enable comprehensive genetic services to be provided countrywide. While the retrospective and current capacity data in this article are based on actual compiled data, the modelled prospective capacity was limited by the assumptions made by Wishnia et al. [44] adjusted for use in this study model. This included:

- The use of approximate/average values for the projected capacity data, which vary in practice.
- There is no comprehensive, definitive dataset of HCP in the country, necessitating a multisource approach for compiling data.
- Data from one year (2023) were used to calculate qualification rates for MG registrars and GC interns, while this may vary year-on-year.
- Training and employments costs for the two HCP cadres are not included.
- Using the total population of the country as the target population prevents specific, stratified capacity requirements for periods across the life course (peri-natal, childhood, adolescence and old age), when there is greater need for MG/GC.

Despite these limitations, the estimates developed for future required capacity serve as a starting point to highlight and address the limited capacity in genetic services in the country.

### 4.6. Recommendations

Opportunities and recommendations of this study are centered around the future establishment of an inclusive, national stakeholder commission/panel to address capacity deficits in genetic services, based on the system in place in the Netherlands [44,51,68,69]. The aims of such a forum would be to develop effective human resource planning for the genetic and CD health of the country, addressing the identified supply-demand gaps and unmet need, including:

- Comprehensively and transparently plan the MG/GC workforce and staffing norms for the coming decades, considering the predicted epidemiologic shifts and ensuring appropriate numbers of posts are allocated. This may be part of a separate healthcare-wide workforce planning body or initiative and should fully consider genetic services in the packages of care offered through NHI.
- Undertaking similar audits for medical scientists, genetic nurses and other associated HCP cadres (e.g., chemical pathologists) within genetic services and for associated specialties across healthcare, to quantify capacity shortfalls. Prospective models could also be stratified for these cadres, as well as MG/GC for specific sub-populations when CD more commonly arise, i.e., paediatric population aged 0–17 years and adults aged 50+ as per the original model for other cadres [44].
- Developing the health economics evidence-base to identify the costs, health outcomes and opportunity costs for components of genetic services, to inform implementation of relevant healthcare policies [21]. An extrinsic, independent approach, incorporating global best practice on Health Technology Assessment is needed to develop a detailed, costed implementation plan for the South African Clinical Guidelines for Genetic Services [21].
- Clarifying and standardising the professional level and renumeration of GC and developing national GC capacity ratios as a component of human resource planning for healthcare.
- Consider reinstating the role and training of genetic nurses countrywide and extending the scope of practice of other relevant HCP, including community healthcare workers, to expand genetic services capacity [70].
- Developing innovative solutions to address identified geographical inequity, such as expanding telehealth countrywide and funding of outreach clinics.
- Undertaking an audit of biochemical/genetic testing available in SA, consolidated into an innovative, updatable, accessible format to facilitate HCP referrals for testing—in-step with emerging technologies, new offerings and initiatives.

## 5. Conclusions

The results of this audit of past, current and modelled future capacity for MG/GC indicate that both cadres are insufficient in number to meet the current and future needs of the population. Almost 30 years after these HCP cadres were first recognized in SA, collective capacity remains at only 10%. On this current trajectory, SA will not meet SDG 3 2030 targets [16] and those affected by, and at risk of CDs, will be “*left behind*”. The majority of these are society’s most vulnerable—children and those living with disability who may remain undiagnosed/misdiagnosed, and die prematurely or live with preventable lifelong disability, unable to access the care they require to optimise quality of life. As the population ages and adult-onset CDs become more prevalent, these collective issues will significantly increase the burden of disease. CDs must be addressed by prioritising this collective in both the current healthcare scenario as well as via NHI and the anticipated increase in health seeking [51]. This requires improved coordination and planning to increase the availability of HCP—necessitating costed implementation plans and allocation of appropriate funding for genetic infrastructure and an expanded, relevant accompanying human workforce.

*“Health workforce planning needs to be actively and continuously managed in order to prevent supply-demand gaps from emerging, as has occurred in South Africa”* [44].

## Figures and Tables

**Table 3:** Number of actual (2024) versus recommended [40,44] medical geneticists (MG) and genetic counsellors (GC) and corresponding capacity percentage by South African (SA) province, for both public and private healthcare sectors.

**SA Province**	**Publicly Served Population (‘000)**	**% of Total Population** **[30]**	**Medical Geneticists:**			**Genetic Counsellors:**		
			**Public**			**Public**		
			Required	Actual	% Capacity	Required	Actual	% Capacity
Eastern Cape	6,160	12%	13	0	0%	52	0	0%
Free State	2,540	5%	5	0	0%	21	0	0%
Gauteng	12,870	24%	27	6	22%	108	6	6%
KwaZulu-Natal	10,795	20%	23	0	0%	91	0	0%
Limpopo	6,096	11%	13	0	0%	51	0	0%
Mpumalanga	4,521	9%	9	0	0%	38	0	0%
Northern Cape	1,066	2%	2	0	0%	9	0	0%
North-West	3,455	7%	7	0	0%	29	0	0%
Western Cape	5,598	11%	12	3	25%	47	4	9%
**SA Total**	**53,102**	**100%**	**112**	**9**	**8%**	**446**	**10**	**2%**
**SA Province**	**Privately Served Population (‘000)**	**% of Total Population** **[30]**	**Medical Geneticists:**			**Genetic Counsellors:**		
			**Private**			**Private**		
			Required	Actual	% Capacity	Required	Actual	% Capacity
Eastern Cape	726	7%	2	0	0%	6	0	0%
Free State	427	4%	1	0	0%	4	1	28%
Gauteng	3581	37%	8	2	27%	30	8	27%
KwaZulu-Natal	1328	14%	3	0	0%	11	2	18%
Limpopo	586	6%	1	0	0%	5	0	0%
Mpumalanga	503	5%	1	0	0%	4	0	0%
Northern Cape	196	2%	0	1	243%	2	0	0%
North-West	537	6%	1	0	0%	5	0	0%
Western Cape	1870	19%	4	1	25%	16	7	45%
**SA Total**	**9752**	**100%**	**20**	**4**	**20%**	**82**	**18**	**22%**

**Table 4:** Number and proportional capacity of medical geneticists and genetic counsellors currently practicing in South Africa (Scenarios 1 and 2) versus HRH recommended capacity (Scenario 3) based on 2024 population [43] with age-range breakdown.

	Public Sector		Private Sector		All Sectors	
Cadre	Current (Required)	Current Capacity	Current (Required)	Current Capacity	Current (Required)	Current Capacity
Medical Geneticists	9 (114 ^a^)	8%	4 (19 ^a^)	22%	13 ^b^ (132)	10%
Genetic Counsellors	10 (455 ^c^)	2%	18 (74 ^c^)	24%	28 ^d^ (529)	5%
**Total**	**19 (568)**	**3%**	**22 (93)**	**24%**	**41 (661)**	**6%**
**Age Range**	**Medical Geneticists**			**Genetic Counsellors**		
**Current Age (years)**	**Number (%)**	**Public**	**Private**	**Number (%)**	**Public**	**Private**
26–30	0	0	0	8 (29%)	4	4
31–35	0	0	0	4 (14%)	3	1
36–40	1 (8%)	0	1	4 (14%)	1	3
41–45	5 (38%)	4	1	3 (11%)	0	3
46–50	1 (8%)	1	0	2 (7%)	0	2
51–55	1 (8%)	0	1	5 (18%)	2	3
56–60	2 (15%)	1	1	2 (7%)	0	2
60+	3 (23%)	3	0	0	0	0
**Total**	**13**	**9**	**4**	**28**	**10**	**18**

^a^ HRH rate of 0.21 MG per 100,000 population [44]. ^b^ Includes 3 males, 10 females. ^c^ Rate of 0.84 GC per 100,000 population (4 GC per 1 MG). ^d^ Includes 1 male, 27 females.

**Table 5:** Number of medical geneticists (MG) and genetic counsellors (GC) currently in-training in South Africa.

Category & Duration	Estimated Completion	UCT	SU *	Wits	Total
MG Registrars	2024	0	0	1	1
	2025	1	0	0	1
	2026	0	1	1	2
	2027	0	0	2	2
	2028	0	0	2	2
	2029	1	0	0	1
**MG Registrars: Sub-total**		**2**	**1**	**6**	**9**
GC Students	2024	3	0	5	8
GC Interns	2024	2	0	0	2
	2025	4	0	2	6
**GC Students/Interns: Sub-total**		**9**	**0**	**7**	**16**

UCT = University of Cape Town, SU = Stellenbosch University, Wits = University of the Witwatersrand; * = SU offers intern training only for GC. MG registrar training is four years and GC internship training is two years.

## Data Availability

All collated data is available either in the article or the Supplementary Table S1, which has been anonymised to comply with local privacy laws. Further inquiries can be directed to the corresponding author. The model’s original Python code was shared under a Data Transfer Agreement with the authors of Wishnia et al. in 2019 [45] who should be contacted for further details.

## Supplementary Materials

The following supporting information can be downloaded at: https://www.mdpi.com/article/10.3390/genes15091173/s1, Table S1: Anonymised list of all medical geneticists and genetic counsellors included in the retrospective analysis.

## References

[1] Modell B. (1992). The need for a science of community genetics. Proceedings of the Genetic Service Delivery.

[2] Modell B., Kuliev A.M., Wagner M. (1991). Community Genetics Services in Europe: Report on a Survey.

[3] Ten Kate L., Al-Gazali L., Anand S., Bittles A., Cassiman J.-J., Christianson A., Cornel M.C., Hamamy H., Kääriäinen H., Kristoffersson U. (2010). Community genetics. Its definition 2010. J. Community Genet..

[4] Modell B., Kuliev A. (1998). The history of community genetics: The contribution of the haemoglobin disorders. Public Health Genom..

[5] WHO Regional Office for the Eastern Mediterranean (1997). Community Control of Genetic and Congenital Disorders.

[6] Boulyjenkov V. (1998). WHO Human Genetics Programme. Community Genet..

[7] Knottnerus J. (2003). Community genetics and community medicine. Fam. Pract..

[8] Al-Gazali L., Alwash R., Abdulrazzaq Y. (2005). United Arab Emirates: Communities and community genetics. Public Health Genom..

[9] World Health Organization Services for the Prevention and Management of Genetic Disorders and Birth Defects in Developing Countries. Proceedings of the Report of a Joint WHO/WAOPBD Meeting.

[10] World Health Organization Primary health care approaches for the control of congenital disorders and disability. In Proceedings of the Report of a WHO Meeting, Cairo, Egypt, 6–8 December 1999. https://apps.who.int/iris/bitstream/handle/10665/66571/WHO_HGN_WG_00.1.pdf?sequence=1&isAllowed=y.

[11] World Health Organization Management of Birth Defects and Haemoglobin Disorders. Proceedings of the Report of a Joint Who-March of Dimes Meeting.

[12] World Health Organization (2011). Community Genetics Services: Report of a Who Consulation on Community Genetics in Low and Middle Income Countries.

[13] Christianson A. (2001). Community genetics in South Africa. Public Health Genom..

[14] United Nations The Millennium Development Goals Report 2015. https://www.un.org/millenniumgoals/2015_MDG_Report/pdf/MDG%202015%20rev%20(July%201).pdf.

[15] World Health Assembly (2010). Sixty-Third World Health Assembly-Resolution 63.17. Birth Defects. http://apps.who.int/gb/ebwha/pdf_files/WHA63/A63_R17-en.pdf.

[16] United Nations Sustainable Development Goal 3: Ensure Healthy Lives and Promote Wellbeing for All and at All Ages. http://www.un.org/sustainabledevelopment/health/.

[17] World Health Assembly (2023). Seventy-Sixth World Health Assembly Resolution 76.19. Accelerating efforts for preventing micronutrient deficiencies and their consequences, including spina bifida and other neural tube defects, through safe and effective food fortification. https://apps.who.int/gb/ebwha/pdf_files/WHA76/A76_R19-en.pdf.

[18] Christianson A., Howson C.P., Modell B. (2006). March of Dimes: Global Report on Birth Defects, the Hidden Toll of Dying and Disabled Children.

[19] World Health Organization Community approaches to the control of hereditary diseases. Proceedings of the Report of a WHO Advisory Group.

[20] Malherbe H.L., Modell B., Blencowe H., Strong K.L., Aldous C. (2023). A review of key terminology and definitions used for birth defects globally. J. Community Genet..

[21] National Department of Health (2021). Clinical Guidelines for Genetic Services.

[22] Malherbe H.L., Christianson A.L., Aldous C., Christianson M. (2016). Constitutional, legal and regulatory imperatives for the renewed care and prevention of congenital disorders in South Africa. S. Afr. J. Bioeth. Law.

[23] Dragojlovic N., Borle K., Kopac N., Ellis U., Birch P., Adam S., Friedman J.M., Nisselle A., Study G., Elliott A.M. (2020). The composition and capacity of the clinical genetics workforce in high-income countries: A scoping review. Genet. Med..

[24] World Bank World Bank Country and Lending Groups. https://datahelpdesk.worldbank.org/knowledgebase/articles/906519-world-bank-country-and-lending-groups.

[25] Statistics South Africa (2022). Statistical Release P0302. Mid-Year Population Estimates 2022.

[26] Statistics South Africa (2023). Statistical Release P0305. Recorded Live Births 2022.

[27] Wessels J., Sherman G., Bamford L., Makua M., Ntloana M., Nuttall J., Pillay Y., Goga A., Feucht U. (2020). The updated South African national guideline for the prevention of mother to child transmission of communicable infections (2019). S. Afr. J. HIV Med..

[28] Johnson L., Meyer-Rath G., Dorrington R., Puren A., Seatlhodi T., Zuma K., Feizzadeh A. (2022). The effect of HIV programs in South Africa on national HIV incidence trends, 2000–2019. J. Acquir. Immune Defic. Syndr..

[29] Dorrington R., Bradshaw D., Laubscher R., Nannan N. (2021). Rapid Mortality Surveillance Report 2019 & 2020.

[30] Statistics South Africa (2023). Statistical Release P0318. General Household Survey 2022.

[31] Republic of South Africa (2024). National Health Insurance, Act 20 of 2023.

[32] Op’t Hof J., Roux J. (1983). Genetic services in the State Health Department of the RSA–development and structure. S. Afr. Med. J..

[33] Jenkins T. (1990). Medical genetics in South Africa. J. Med. Genet..

[34] Republic of South Africa (2004). National Health Act No. 61.

[35] Kromberg J.G., Sizer E.B., Christianson A.L. (2013). Genetic services and testing in South Africa. J. Community Genet..

[36] Kromberg J., Krause A. (2013). Human genetics in Johannesburg, South Africa: Past, present and future. S. Afr. Med. J..

[37] Beighton P., Fieggen K., Wonkam A., Ramesar R., Greenberg J. (2012). The University of Cape Town’s contribution to medical genetics in Africa: From the past into the future. S. Afr. Med. J..

[38] Wessels T., Greenburg J., Fourie K., Kromburg J. (2024). Genetic Counseling in South Africa: A growing profession. Genet. Med..

[39] National Department of Health (2001). Human Genetics Policy Guidelines for the Management and Prevention of Genetic Disorders, Birth Defects and Disabilities.

[40] National Department of Health (2005). National Guidelines for the Care and Prevention of the Most Common Genetic Disorders Birth Defects and Disabilities.

[41] Malherbe H.L., Aldous C., Christianson A. (2015). Need for services for the care and prevention of congenital disorders in South Africa as the country’s epidemiological transition evolves. S. Afr. Med. J..

[42] Baker L. (2010). The South African expanded programme on immunisation schedule: Vaccinology. Prof. Nurs. Today.

[43] National Department of Health (2020). 2030 Human Resources for Health Strategy: Investing in the Health Workforce for Universal Health Coverage.

[44] Wishnia J., Strugnell D., Smith A., Ranchod S. (2019). The Supply of and Need for Medical Specialists in South Africa. https://percept.co.za/wp-content/uploads/2019/10/The-supply-of-and-need-for-medical-specialists-in-SA-PERCEPT.pdf.

[45] Pyeritz R.E., Korf B.R., Grody W.W. (2018). Emery and Rimoin’s Principles and Practice of Medical Genetics and Genomics: Foundations.

[46] Rimoin D.L., Victor A. (2008). McKusick 1921–2008. Nat. Genet..

[47] Kromberg J.G., Wessels T.-M., Krause A. (2013). Roles of genetic counselors in South Africa. J. Genet. Couns..

[48] Johnson L.F., Dorrington R.E., Moolla H. (2017). Progress towards the 2020 targets for HIV diagnosis and antiretroviral treatment in South Africa. S. Afr. J. HIV Med..

[49] Modernisation of Tertiary Services Project Team (2003). Strategic Framework for the Modernisation of Tertiary Hospital Services. http://www.kznhealth.gov.za/hospmodernisation.pdf.

[50] Dacre J., Shepherd S. (2010). Women and medicine. Clin. Med..

[51] Smith A., Wishnia J., Strugnell D., Ranchod S. (2018). Human resources for health planning and National Health Insurance: The urgency and the opportunity. S. Afr. Health Rev..

[52] Gxobole A., Naidoo R., Singh B. (2023). The supernumerary registrar experience in KwaZulu-Natal. S. Afr. J. Surg..

[53] Nippert I., Christianson A., Gribaldo L., Harris H., Horovitz D., Abdel-Raouf R.K., Kent A., Kristoffersson U., Padilla C.D., Penchaszadeh V. (2013). Genetic Testing in Emerging Economies (GenTEE). Summary Report.

[54] Rudolfson N., Lantz A., Shrime M.G., Johnson W., Smith M.D., Hagander L. (2023). South Africa and the Surgical Diaspora-A Hub for Surgical Migration and Training. World J. Surg..

[55] National Treasury South African Government Treasury on Guidelines on Cost Containment Measures. https://www.gov.za/news/media-statements/treasury-guidelines-cost-containment-measures-18-sep-2023.

[56] Khan S., Ntatamala I., Baatjies R., Adams S. (2024). Prevalence and determinants of burnout among South African doctors during the COVID-19 pandemic. S. Afr. J. Psychiatry.

[57] Statistics South Africa (2023). Census 2022 (Statistical Release P0301.4).

[58] Malherbe H., Christianson A., Woods D., Aldous C. (2017). The case for the genetic nurse in South Africa. J. Community Genet..

[59] Omran A. (1971). The epidemiologic transition: A theory of the epidemiology of population change. Milbank Mem. Fund..

[60] Solanki G., Kelly G., Cornell J., Daviaud E., Geffen L. (2019). Population ageing in South Africa: Trends, impact, and challenges for the health sector. S. Afr. Health Rev..

[61] Strong K.L., Requejo J., Agweyu A., Billah S.M., Boschi-Pinto C., Horiuchi S., Jamaluddine Z., Lazzerini M., Maiga A., McKerrow N. (2021). Revitalizing child health: Lessons from the past. Glob. Health Action.

[62] Perin J., Mai C.T., De Costa A., Strong K., Diaz T., Blencowe H., Berry R.J., Williams J.L., Liu L. (2023). Systematic estimates of the global, regional and national under-5 mortality burden attributable to birth defects in 2000–2019: A summary of findings from the 2020 WHO estimates. BMJ Open.

[63] Strong K., Robb-McCord J., Walani S., Mellado C., Botto L.D., Lay-Son G., Diaz T., Banu T., Lakhoo K., Banerjee A. (2024). Action against birth defects: If not now, when?. Glob. Health Action.

[64] Department of Health (2021). South African Maternal, Perinatal and Neonatal Health Policy.

[65] National Department of Health (2022). The National Strategic Plan for the Prevention and Control of Non-Communicable Diseases, 2022–2027.

[66] Malherbe H.L., Woods D.L., Aldous C., Christianson A.L. (2016). Review of the 2015 Guidelines for Maternity Care with relevance to congenital disorders. S. Afr. Med. J..

[67] Gomes M., Malherbe H.L. (2024). The impact of COVID-19 on patients impacted by rare diseases and congenital disorders in South Africa—A scoping review. S. Afr. Med. J..

[68] Van Greuningen M., Batenburg R.S., Van der Velden L.F. (2012). Ten years of health workforce planning in the Netherlands: A tentative evaluation of GP planning as an example. Hum. Resour. Health.

[69] Van Greuningen M. Health Workforce Planning in the Netherlands: How a Projection Model Informs Policy Regarding the General Practitioner and Oral Health Care Workforces. https://www.researchgate.net/profile/Malou-Van-Greuningen/publication/309111392_Health_workforce_planning_in_the_Netherlands_how_a_projection_model_informs_policy_regarding_the_general_practitioner_and_oral_health_care_workforces/links/57ff9f1f08ae6fc7fc64f574/Health-workforce-planning-in-the-Netherlands-how-a-projection-model-informs-policy-regarding-the-general-practitioner-and-oral-health-care-workforces.pdf.

[70] Perry H.B., Zulliger R., Rogers M.M. (2014). Community health workers in low-, middle-, and high-income countries: An overview of their history, recent evolution, and current effectiveness. Ann. Rev. Public Health.

