# Supplementary material for: A Capacity Audit of Medical Geneticists and Genetic Counsellors in South Africa, 2024: A National Crisis
Source: Genes (Basel). 2024 Sep 6;15(9):1173. doi: 10.3390/genes15091173 (PMC11430864; doi:10.3390/genes15091173)
Supplement: Supplementary file 1 [file genes-15-01173-s001.zip › genes-3147228-supplementary.pdf]

Supplementary file

Table S1: Anonymised list of all medical geneticists and genetic counsellors included in the retrospective analysis.

MEDICAL GENETICISTS							
**No**	**Registration via**	**HPCSA Reg Year as MG**	**PractisingY/N**	**If Y - pvt or state If N - retired, emigrated, researcher, academia**	**% private**	**% state**	**Province (SA)**
1	Grandfather Clause 1	1997	N	Emigrated			Belgium
2	Grandfather Clause 1	1997	N	Passed away			Passed away
3	Grandfather Clause 1	1997	Y	Emigrated			Australia
4	Grandfather Clause 1	1997	N	Passed away			Passed away
5	Grandfather Clause 1	1997	N	Retired			Cape Town
6	Grandfather Clause 1	1997	N	Academia			Gauteng
7	Grandfather Clause 1	1997	Y	Retired	0	0	Western Cape
8	Grandfather Clause 1	1997	N	Retired			Retired
9	Grandfather Clause 1	1997	N	Passed away			Passed away
10	Grandfather Clause 1	1997	Y	Retired			Western Cape
11	Grandfather Clause 1	1998	Y	Retired			Western Cape
12	Grandfather Clause 1	1998	Y	Retired			Western Cape
13	Grandfather Clause 1	1998	N	Retired			Retired
14	SS training	2003	Y	State	5	95	Gauteng
15	SS training	2003	Y	Emigrated			New Zealand
16	SS training	2005	Y	State	0	100	Western Cape
17	SS training	2005	Y	Emigrated			Australia
18	SS training	2006	Y	Private	100	0	Gauteng
19	SS training	2006	Y	Emigrated			Canada
20	SS training	2006	Y	State			Gauteng
21	SS training	2008	N	Passed away			Passed away
22	Grandfather Clause II	2008	Y	State	10	90	Gauteng
23	Grandfather Clause II	2008	Y	Private	100	0	Gauteng
24	Grandfather Clause II	2011	N	Emigrated			USA
25	Grandfather Clause II	2012	N	Academia	0	0	Gauteng
26	4 year specialty	2012	Y	State	0	100	Western Cape
27	4 year specialty	2013	Y	Emigrated			New Zealand
28	4 year specialty	2013	Y	State	5	95	Gauteng
29	4 year specialty	2014	Y	Emigrated			Ireland
30	4 year specialty	2016	Y	Emigrated			Switzerland
31	4 year specialty	2016	Y	Emigrated			Australia
32	4 year specialty	2018	Y	Private	100	0	Northern Cape
33	4 year specialty	2019	Y	State	1	99	Western Cape
34	4 year specialty	2019	Y	State	10	90	Gauteng
35	4 year specialty	2019	Y	Emigrated			England
36	4 year specialty	2021	Y	Emigrated			Oman
37	4 year specialty	2021	Y	Private	100	0	Western Cape
38	4 year specialty	2021	Y	Emigrated			Canada
39	4 year specialty	2022	Y	State	5	90	Gauteng
40	4 year specialty	2024	N	Emigrated			Ireland
41	4 year specialty	2024	N	Not practicing			Gauteng

GENETIC COUNSELLORS						
No	HPCSA Reg Year	Practicing Y/N	If Y - pvt or state If N - retired, emigrated, researcher	% private	% state	Province (SA)
1	1995	N	Passed away			
2	1996	N	Retired			
3	1995	N	Retired			Western Cape
4	1998	Y	Private	100	0	Gauteng
5	1998	Y	State	0	100	Western Cape
6	2001	N	Retired	0	0	Western Cape
7	2005	N	Research	0	0	Gauteng
8	2006	N	Emigrated			Netherlands
9	2006	Y	Private	100	0	KZN
10	2007	N	Emigrated			Australia
11	2007	Y	Private	100	0	Western Cape
12	2007	Y	Private	100	0	Gauteng
13	2008	Y	Private	100	0	Gauteng
14	2009	N	Research	0	100	Western Cape
15	2009	Y	Private	100	0	Gauteng
16	2009	N	Research	0	0	Western Cape
17	2009	Y	State	20	80	Western Cape
18	2009	N	Emigrated			Australia
19	2009	N	Emigrated			Oman
20	2010	N	Emigrated			New Zealand
21	2011	Y	Private	100	0	Gauteng
22	2013	Y	State	0	100	Western Cape
23	2013	Y	Private	100	0	Western Cape
24	2013	N	Research	0	0	Canada
25	2014	Y	Private	100	0	Gauteng
26	2014	Y	Private	100	0	Gauteng
27	2014	Y	Private	100	0	Gauteng
28	2016	Y	Private	100	0	Western Cape
29	2016	N	Emigrated			Singapore
30	2017	Y	State	5	95	Gauteng
31	2017	N	Emigrated			England
32	2019	Y	State	20	80	Gauteng
33	2019	N	Emigrated			England
34	2019	N	Emigrated			England
35	2019	Y	Private	100	0	Western Cape
36	2019	Y	State	0	100	Western Cape
37	2019	Y	State	10	90	Gauteng
38	2019	N	Research	5	0	Western Cape
39	2019	Y	Private	100	0	Freestate
40	2020	Y	State	10	90	Gauteng
41	2020	Y	Private	60	40	Western Cape
42	2020	N	Emigrated			England
43	2021	Y	Private	100	0	Western Cape
44	2021	N	Emigrated			England
45	2021	Y	State	10	90	Gauteng
46	2021	N	Emigrated			England
47	2022	Y	Private	90	10	Western Cape
48	2022	N	Emigrated			England
49	2022	N	Emigrated			England
50	2022	N	Emigrated			Ireland
51	2023	Y	Private	100	0	KZN
52	2023	Y	State	0	100	Gauteng
53	2023	N	Emigrated			Singapore
54	2024	N	Laboratory	0	0	Western Cape
